# The Swiss Spinal Cord Injury Cohort Study (SwiSCI) biobank: from concept to reality

**DOI:** 10.1038/s41393-024-00958-x

**Published:** 2024-01-29

**Authors:** Ramona M. Zeh, Marija Glisic, Simona Capossela, Alessandro Bertolo, Ezra Valido, Xavier Jordan, Margret Hund-Georgiadis, Jürgen Pannek, Inge Eriks-Hoogland, Gerold Stucki, Jivko Stoyanov

**Affiliations:** 1 Swiss Paraplegic Research, Guido A. Zäch Str. 4, 6207 Nottwil, Switzerland; 2 Institute of Social and Preventive Medicine (ISPM), University of Bern, Bern, Switzerland; 3 Department of Orthopedic Surgery, University of Bern, Bern Inselspital, Bern, Switzerland; 4 Clinique romande de réadaptation, Avenue du Grand-Champsec 90, 1950 Sion, Switzerland; 5 REHAB Basel, Im Burgfelderhof 40, 4055 Basel, Switzerland; 6 Swiss Paraplegic Center, Guido A. Zäch Str. 1, 6207 Nottwil, Switzerland; 7 Inselspital, Bern University Hospital, University of Bern, Bern, Switzerland

**Keywords:** Translational research, Diagnostic markers

## Abstract

**Objectives:**

To describe the concept, establishment and the operationalization of the biobank of the Swiss Spinal Cord Injury Cohort Study (SwiSCI), the available biosamples, and demographic and clinical characteristics of study participants.

**Setting:**

The SwiSCI biobank is a platform for research within SwiSCI. It collects and processes serum, plasma, PBMCs, RNA, DNA, and urine from three rehabilitation centers. Samples are collected at admission to first rehabilitation and at discharge. Additionly, the biobank provides services to projects nested in SwiSCI or otherclinical trials among Spinal Cord Injury population.

**Methods:**

Descriptive statistics were used for an overview of available biosamples, study participant characteristics, and comparison of the participating centers.

**Results:**

Between the SwiSCI biobank establishment on June 27th, 2016, and October 19th, 2023, the SwiSCI Study has obtained informed consent from 524 individuals. Of these, 315 (60.1%) have agreed to donate biospecimens to the biobank. The average age of the contributors was 54 years (range: 38–65), with the majority being male (80%). Most participants suffered from traumatic injuries (66%) and were classified as paraplegic (64%). Approximately 80% presented with motor and sensory-incomplete SCI. The median Spinal Cord Independence Measure (SCIM) score was 31 (Interquartile Range: 19–58). The proportion of individuals providing paired biosamples at two distinct time points ranged from 63% (for RNA) to 65% (for urine and urine sediment).

**Conclusions:**

The SwiSCI biobank is a unique platform designed to serve as a basis for collaborative SCI research, including multi-omics approaches. The longitudinal collection of biospecimens and cryopreservation of multiple aliquots for each participant are fundamental for scrutinizing the temporal associations, ensuring research reproducibility, and achieving an adequate sample size for future investigations.

## Introduction

Spinal cord injury (SCI) is relatively rare condition, which incidence rate globally was estimated to be on average 13 (11–16) per 100,000 population [1]. The injury causes impairment or loss of sensory and/or motor function below injury level, leads to a lifelong disability and affects overall health and quality of life of SCI individual, thus, posing a tremendous burden to the health of the affected individuals as well as to the healthcare system [2, 3].

The first research article addressing SCI has been published by Dr. John Gordon, a fellow of the Royal College of Surgeons in Edinburgh. The article was published in *Edinburgh Medical and Surgical Journal* in October 1817, and it one of the first case reports describing development and acute treatment of SCI [4]. SCI research for the next 150 years has been mostly focusing on acute management, assistive technologies and neurological recovery in SCI individuals. With improvement in acute care and prolonged life-expectancy over the past three decades, SCI research has now also to focus on treatment of secondary health conditions (pain, pressure ulcers, urinary tract infections, and cardiometabolic diseases). State of the art “omics” research and applications of artificial intelligence and machine learning start to contribute to understanding neurological recovery and functioning post-injury [5]. Despite these tremendous improvements in research methodology, there are unique challenges for research in SCI individuals and these comprise the following: (i) small/insufficiently powered single-center studies (both, observational and interventional studies); (ii) limited methodological quality (e.g., cross-sectional studies, evident selection bias, lack of adjustment for confounders in statistical analyses, etc.); (iii) predominance of studies conducted among individuals with chronic SCI; (iv) intentional exclusion of women in SCI research to make study populations more homogenous; (v) limited number of studies conducted among individuals with non-traumatic injury [6].

Collaborative research (e.g., international consortia or multi-centric studies) is going to be the key to generate epidemiologically sound data in SCI field. Furthermore, the access to human biological material and the associated clinical data are crucial for clinical and translational research in SCI, to test the hypotheses generated by association studies. Considering the rarity of the injury, biobanks may serve this purpose by collecting and organizing biosamples in a systematic way for their distribution to scientists engaged in research on SCI [7]. This article covers the concept and the operationalization of the establishment of a biobank anchored to the multi-center Swiss Spinal Cord Injury Cohort Study (SwiSCI).

## Methods

### Setting of the SwiSCI cohort and study design

The focus of this article is the participation of the biobank in the inception cohort of the SwiSCI study. The SwiSCI biobank provides a framework for conducting research projects nested within the SwiSCI cohort study. Detailed information on the SwiSCI study design and collected data can be found elsewhere [8, 9]. In brief, SwiSCI is established as prospective cohort study in collaboration between four major rehabilitation centers across Switzerland that serve as regional catchment areas for individuals requiring specialized therapy for SCI. Individuals with an SCI attributable to a congenital condition, neurodegenerative disorder, or Guillain–Barré syndrome, or who had a new SCI in the context of palliative care, were not invited to participate. A wide range of demographic, biopsychosocial, clinical parameters and biological samples from persons newly diagnosed with traumatic (TSCI) or non-traumatic (NTSCI) SCI receiving primary specialized rehabilitation were prospectively collected.

Data acquisition commenced on 1st May 2013 for inpatients and was extended to outpatient settings starting 1st August 2016. Data are collected at four distinct post-diagnosis time-points: T1 (28 days, range: 16–40 days), T2 (84 days, range: 70–98 days), T3 (168 days, range: 150–186 days), and T4 (0–15 days prior to discharge). Among the rehabilitation centers, three contribute to routine SwiSCI biobank sampling, while the fourth—University Hospital Balgrist—engages in project-specific biosampling.

Blood and urine serve as the primary biospecimens earmarked for long-term storage due to their minimally invasive (blood) or non-invasive (urine) collection methods. This aligns well with routine clinical practices, allowing for easy integration of biobank biosampling. Other clinically pertinent data such as MRI findings may be incorporated into future studies as they are stored in a centralized clinical database.

**Fig. 1:**
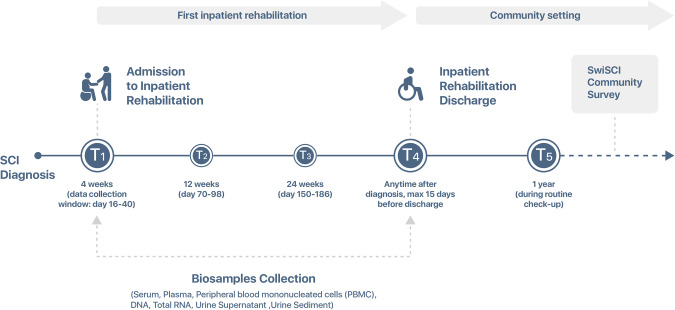
Schematic representation of longitudinal data and biosample collection in the SwiSCI Inception Cohort. This figure delineates the timeline and specific time points (T1 to T4) at which both clinical data and biological samples were collected within the context of the SwiSCI inception cohort study.

Biobank sampling is conducted at time points T1 (admission to rehabilitation) and T4 (discharge from rehabilitation), Fig. 1. These time points are consistent with the general SwiSCI assessment schedule. Upon consenting to participate in the SwiSCI study, patients undergo routine blood sampling for cardiovascular risk factors at these time-points to minimize patient burden.

Biobank sampling was initiated on 27th June 2016 in the SPZ in Nottwil, followed by REHAB Basel Klinik für Neurorehabilitation und Paraplegiologie and CRR Suva (Clinique romande de réadaptation) on 23rd August 2018 and 15th January 2019. Funding for the SwiSCI biobank is provided by the Swiss Paraplegic Foundation.

### The SwiSCI biobank services as infrastructure

Additionally, to the core use as a SwiSCI biobank, in 2021, the SwiSCI biobank Infrastructure was founded and also certified by Swiss Biobanking Platform (SBP), allowing to collect, process and store biosamples from other studies, connected or not to SwiSCI. At the moment, the SwiSCI biobank Infrastructure collects or has collected biosamples for three additional studies: UROVAXOM, TASCI and Athletic-probiotic study. Serum, plasma, peripheral blood mononuclear cells (PBMC), RNA, DNA, urine and urinary microbiome biosamples are being collected within the UROVAXOM study, which is a pilot clinical trial that aims to explore the role of immunomodulation for primary prevention of urinary tract infections in patients with SCI [10]. Within the TASCI study, a randomized prospective clinical trial aimed to explore the efficacy of transcutaneous tibial nerve stimulation in preventing neurogenic detrusor overactivity, serum, plasma, PBMC, RNA, DNA, urine, urinary and gut microbiome biosamples are prospectively processed and stored [11]. Finally, we have recently finalized collection of serum, plasma and gut microbiome biosamples within the pilot randomized clinical trial which aimed to assess the feasibility of probiotic and prebiotic intervention in athletes with SCI (the Athletic study) [12].

### Ethics, governance and management of the biobank

The SwiSCI biobank ethical approval was given on 2nd November 2015 by the Ethikkommission Nordwest- und Zentralschweiz (EKNZ). The biobank has a clearly defined governance and organizational structure. The head of the biobank is responsible for strategic management and collaborations and has the overall responsibility for operating the biobank. Administration and operational management are done by the operational manager who ensures the day-to-day functioning, compliance to the regulations and supervision of technical assistants.

The SwiSCl Study Center coordinates the research assistants in the participating centers. Responsibility of the anonymisation management is with the clinics. The key fiduciary manages the key according to a defined procedure, de-coupling privacy-related information from the corresponding downstream samples and data.

**Fig. 2:**
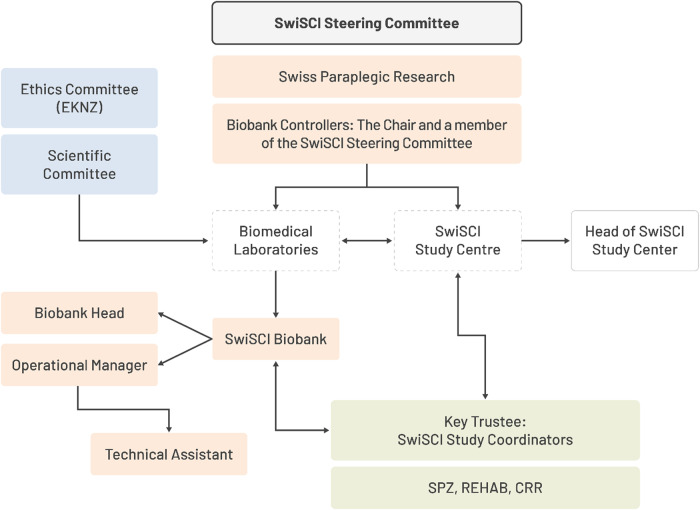
Organizational structure of the SwiSCI biobank. The organigram illustrates the hierarchical and functional architecture of the SwiSCI biobank, showcasing the relationship between various stakeholders, departments, and processes integral to its operations.

The SwiSCI study is overall monitored and controlled by the SwiSCI steering committee. The biobank controllers are responsible for the monitoring and coordination of the SwiSCI biobank. The head of the biobank reports to the biobank controllers and the steering committee. An organigram of the biobank shows the responsibilities and involved parties (Fig. 2).

### Recruitment procedure

Potential study participants are recruited at SwiSCI centers by trained research assistants. The informed consent procedure is standardized between the centers and is performed just before or during early rehabilitation. No consent is collected in emergency contexts such as the acute surgery phase but afterwards. Consent can be withdrawn at any time. In the event of withdrawal, all biosamples and samples belonging to that participant are to be destroyed. The neurological status of the participants was described using theASIA/ISCoS International Standards for Neurological Classification of Spinal Cord Injury (ISNCSCI) [13].

### Biospecimen collection

All reception and processing steps are formalized in standard operating procedures (SOPs). Peripheral whole blood is collected by trained clinical staff. The phlebotomy procedure uses a butterfly clip (BD) and is standardized across the participating centers. Peripheral whole blood is collected in several formats; (a) monovettes that contain silica clotting factor (Sarstedt), monovettes containing K3 EDTA (Sarstedt), and PAXGene RNA stabilization vacutainers (BD). Silica clotted blood is processed into serum. K3 EDTA blood processed into plasma, genomic DNA (gDNA) and PBMC. PAXGene tubes are processed into total RNA (including miRNA). Urine is collected in standard urine monovettes (Sarstedt). Urine is centrifuged, the supernatant is sterile filtered and stored as urine while the pellet is stored as urine sediment. For the TASCI study, immediately after collection the urine is kept on ice until processing. As the TASCI study requires urine purines, 2 mL of the collected urine is boiled shortly. Feces (for TASCI and Athletic study) are collected in gut microbiome DNA collection tubes (OMNIGENE). For more details see Table 1.

**Table 1:** Overview of currently available biosamples.

Biosample	Study				Biobanking procedure			
	SwiSCI	TASCI	UROVAXOM	Athletic study	Biospecimen	Collection Volume	Storage Temp	Storage volume/amount
Serum	+	+	+	+	Whole blood with silica clotting factor	Up to 2 × 7.5 mL blood (15 mL total) For TASCI: Up to 3 × 7.5 mL blood (21.5 mL total) For AC: Up to 1 × 7.5 mL blood (7.5 mL total)	−80°C	0.5 mL
Plasma	+	+	+	+	K3 EDTA whole blood	Up to 3 × 7.5 mL blood (21.5 mL total) For AC: Up to 1 × 7.5 mL blood (7.5 mL total)	−80°C	0.5 mL
Peripheral blood mononuclear cells (PBMC)	+	+	+	NA	K3 EDTA whole blood	Up to 3 × 7.5 mL blood (21.5 mL total)	−150°C	2.5 × 10^6^ cells in 0.5 mL/5.0 × 10^6^ cells in 1.0* mL freezing media
DNA	+	+	+	NA	K3 EDTA whole blood	Up to 3 × 7.5 mL blood (21.5 mL total)	−80°C	1 µg
Total RNA	+	+	+	NA	PAXgene blood	Up to 2 × 2.5 mL blood (5 mL total)	−80°C	500 ng/1 µg**
Urine supernatant	+	+	+	NA	Urine	Up to 3 × 10 mL urine (30 mL total)	−80°C	1.9 mL
Urine sediment	+	+	+	NA	Urine	Up to 3 × 1mL urine (3 mL total)	−80°C	1 mL
Urinary microbiome	NA	+	NA	NA	Urine	2 mL boiled urine (45 sec at 99°C)	−80°C	0.2 mL
Gut microbiome	NA	+	NA	+	Stool	Up to 1.5 mL in stabilization solution	−80°C	0.25 mL

Peripheral whole blood is collected using a butterfly clip in K3-EDTA monovettes (Sarstedt), monovettes with silica clotting factor (Sarstedt) and PAXGene RNA vacutainers (BD). The monovettes and vacutainers are immediately inverted post collection several times. Urine is collected from the catheter in 10 mL urine monovettes (Sarstedt). Feces are collected in gut microbiome DNA collection tube containing 1 mL stabilizing fluid (OMNIgene).

*1 mL storage vial for SwiSCI since 2023.

**40  ng/µL since 2023.

The research assistants in the centers retrieve the monovettes and vacutainers from the clinical wards and remove any identifying information before re-labeling with anonymized IDs (SwiSCI ID or TASCI ID); Infrastructure projects follow the instruction of the study principal investigator (PI). The primary biomaterials collected in SPZ Nottwil are directly handed over to the biobank staff. Biospecimen from REHAB Basel are shipped by express courier to Nottwil and processed/stored by SwiSCI biobank. CRR processes the biospecimen on their own using the same SOPs used in the SwiSCI biobank; frozen samples are shipped to Nottwil on dry ice.

After arrival, SwiSCI biobank personnel assess the fresh biospecimen sets from SPZ or REHAB and uses time stamps (i.e. on collection times, arriving times), volumes and issues among other parameters on sample summary and for REHAB reception sheets. The arrival of the already processed and frozen samples from CRR is also documented on a reception form.

### Biosamples processing

Apart from the SOP for PBMC processing and isolation, feces collection and urine purine storage, all SOPs are derived from and comply with CEN/TS standards for Isolated RNA (CEN/TS 16835-1), Isolated DNA (CEN/TS 16835-2) and urine, venous blood serum and plasma (CEN/TS 16945).

Collection volumes, storage temperatures and storage volumes/amounts can be found in Table 1. For storage, four different types of Azenta Life Sciences tubes are used: 1.9 mL tri-coded tube, 48-format, external thread (with screw cap), with a maximal working volume of 1.9 mL; 1.0 ml tri-coded tube, 96-format, external thread (with screw cap), with a maximal working volume of 1.0 mL; 0.5 mL tri-coded tube, 96-format, external thread (with screw cap), with a maximal working volume of 0.52 mL; and 0.26 mL dual-coded tube, 96-format, external thread (with screw cap), with a maximal working volume of 0.26 mL. A camera-based reader for SBS racks is used for scanning the aliquots into the database and later for fast identification of the SBS-format racked, 2D-coded tubes. The Biobank is managed with the FreezerPro® Sample Management System (Azenta US, Inc).

Detailed SOPs are created for each biobanking process. In brief, after clotting for 30 min at room temperature (RT), the serum monovettes are centrifuged at 1500 x g for 15 min, acceleration and brake speed at 9. The supernatant is aliquoted and stored at −80°C. The urine monovettes are centrifuged at 3000 x g for 5 min at 4°C. The supernatant is pipetted down to the 1 mL mark and pooled before filtration through a 0.22 µm membrane. The remaining 1 mL fractions in the monovettes are vortexed and pooled to generate the urine sediment fraction. The filtered urine supernatant is aliquoted, while the urine sediment is stored as a separate aliquot. All urine fractions are frozen at −80°C. For DNA extraction, 1 mL of K3 EDTA blood is taken directly from one monovettes. Genomic DNA is isolated using the GeneJet kit according to the manufacturer’s instructions and aliquots are stored at −80°C.

The rest of the K3 EDTA blood is gently decanted into a Leukosep tube containing 15.5 mL Lympholyte® Cell Separation density gradient centrifugation media (CEDARLANE). The tube is centrifuged for 20 min at 800 x g, acceleration speed 4, brake speed 0. The plasma layer is pipetted down to approximately 1 cm above the buffy coat, and aliquots are stored at −80°C. The buffy coat is gently aspirated, mixed with complete RPMI (cRPMI) and pelleted at 300 x g for 10 min (acceleration and brake speed 9). The pellet is resuspended in 10 mL cRPMI. Cells are counted using colter method (Scepter Millipore) in addition to a viability assessment with 0.4% Trypan Blue. After a centrifugation at 300 x g for 10 min (acceleration and brake speed 9), the pellet is resuspended in ice cold FBS to achieve a concentration of 10 × 10^6^ PBMC/mL. 2.5 × 10^5^ cells are assayed in triplicate in an alamar blue metabolic assay to establish metabolic activity. The remaining PBMC are diluted to 5 × 10^6^ PBMC/mL with drop by drop addition of ice cold 2X freezing media composed of 20% dimethyl sulfoxide (DMSO) and 80% cRPMI. Cells are either aliquoted in 500 µL (2.5 × 10^6^ PBMC) or 1 mL (5.0 × 10^6^ PBMC) samples. Mr. Frosty containers for rate limited cooling at −1°C/min are used in a −80 °C freezer before storing at −150 °C freezer.

RNA is isolated from 5 mL of peripheral whole blood stabilized in PAXGene RNA vacutainers (BD), using the miRNA isolation kit (Qiagen) according to the manufacturer’s instructions and aliquots are stored at −80 °C. Feces are collected in fecal stabilization tubes (OMNIGENE), homogenized, stored at −80 °C.

Sample processing has to stick exactly to the previously validated and approved SOPs. Every deviation can cause variance in later experiments and therefore has to be noted on the work lists and in the database to be trackable. Time stamps and involved persons have to be noted. SOP version changes are recorded as well as the information which version was used per patient and donation time point.

### Data management

The SwiSCI data derived from patients is coded by an independent unique identifier with a neutral number from the point of view of the researcher. SwiSCI Study and the SwiSCI biobank data are kept in secure web-based SQL database, which is protected by passwords and have daily back-up. The SwiSCI study center also tracks changes to their databases and the identity of the person who accesses them. Biosample handling is performed only with encrypted samples, which are transferred for processing and cryopreservation the SwiSCI biobank according to validated workflows and standard operational procedures. Researchers can apply for SwiSCI data and samples by submitting a research proposal to the Study Center (swisci.research@paraplegie.ch) that undergoes an internal peer review process. Upon approval of the research proposal, pseudonymized data are provided to researchers for analysis. Samples for analysis are provided after an additional approval by the responsible ethics committee.

### Statistical analyses

This paper reports on data collected in the inpatient setting during the period between 27th June 2016 and 19th October 2023. We summarized the baseline characteristics of our study population using mean (and standard error), median (and interquartile range) or counts (and percentages) as applicable. We used the Shapiro-Wilk test to determine the normality of the distribution of SwiSCI study participants characteristics. Kruskal-Wallis signed-rank test or Chi-squared test were used to determine differences in demographic and clinical profiles between SwiSCI study participants collected through three recruitment centers (SPZ, REHAB and CRR). All analyses were performed using Stata 16.1 (StataCorp LLC, College Station, TX) for Windows. All computations were done using two-tailed tests, and a *p*-value of <0.05 was considered statistically significant.

## Results

### Overview of cryopreserved biosamples

**Fig. 3:**
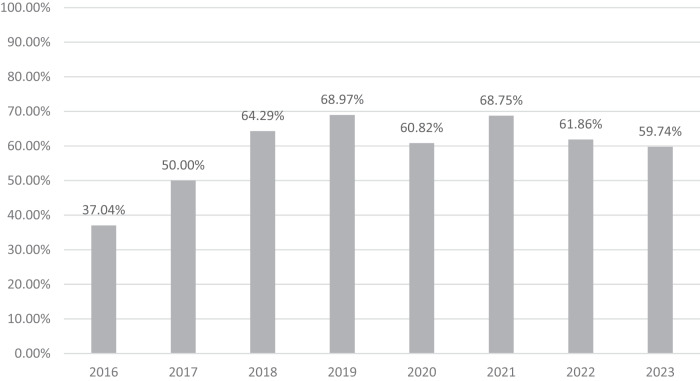
Temporal trends in participation rates in the SwiSCI biobank. This figure presents the trend in participation rates in the SwiSCI biobank from its inception in 2016 to 2023. The graph highlights the stability of the participation rate, which has remained around 60% since 2018. Analysis is based on 315 individuals who agreed to provide their samples to the SwiSCI biobank by 19th October 2023.

From the inception of the SwiSCI biobank on June 27th 2016, to October 19th 2023, a total of 524 individuals consented to participation in the SwiSCI study. Of these, 315 (60.1%) agreed to contribute biosamples to the Biobank. Only two withdrawals of consent have been recorded to date. Figure 3 illustrates a consistent participation rate of approximately 60% since 2018. At the initial rehabilitation phase (T1), plasma, serum, PBMCs, DNA, and urine biosamples were available from over 89% of consenting individuals. RNA samples were available from 87.30% of these participants. By the time of discharge (T4), the proportion of available biosamples reduced to approximately 72%. Of the 315 participants, 195 (61.9%) contributed complete biosamples at both time points (T1 and T4). The percentage of paired biosamples ranged from 62.90% (RNA) to 64.76% (urine and urine sediment). An updated inventory of biosamples, as of October 19th 2023, is presented in Table 2.

**Table 2:** Overview of currently available samples.

Sample type	Number of individuals with available samples at T1	Number of individuals with available samples at T4	Number of individuals with available paired samples (T1 & T4)*
Plasma	282 (89.52%)*	228 (72.38%)	202 (64.13%)
Serum	287 (91.11%)	228 (72.38%)	201 (63.81%)
PBMC, peripheral blood	282 (89.52%)	228 (72.38%)	202 (64.13%)
DNA, peripheral whole blood	281 (89.21%)	227 (72.06%)	202 (64.13%)
Total RNA, peripheral whole blood	275 (87.30%)	227 (72.06%)	195 (62.90%)
Urine	284 (90.16%)	228 (72.38%)	204 (64.76%)
Urine sediment	284 (90.16%)	228 (72.38%)	204 (64.76%)

*(% of total donor number); calculation based on 315 individuals as of 19th October 2023.

Contributors to missing samples included short inpatient stays (*n* = 39), Covid-19 restrictions (*n* = 9), participant refusal (*n* = 7), transfer to a different hospital (*n* = 4), delayed consent (*n* = 4), and mortality (*n* = 3). Additionally, 23 participants were inpatients at the time this report was prepared.

### Demographics of SwiSCI biobank participants

Table 3 delineates the key characteristics of individuals whose biosamples are stored in the SwiSCI biobank. The majority of participants experienced a traumatic SCI (65.71%), were diagnosed with paraplegia (63.87%), and exhibited motor and sensory-incomplete SCI (79.74%). The median SCIM score stood at 31 (IQR 19–58), with a median age of 54 years (38–65). Most participants were male (80.19%). Notable disparities in age, prevalence of motor complete injuries, and SCIM scores were observed across the three contributing rehabilitation centers, potentially reflecting the specialized focus of SPZ on SCI rehabilitation, in contrast to the broader service provision of REHAB Basel and CRR Sion.

**Table 3:** Personal and clinical characteristics of individuals who provided consent to the SwiSCI biobank.

Characteristics	Total study population (*N* = 317)	SPZ (*N* = 186)	REHAB (*N* = 48)	CRR (*N* = 81)	*P value*
Age, years, median (IQR)^1^	54 (38–65)	51 (36–64)	60.5 (49–73)	55 (43–63)	**0.01**
Education years, median (IQR)^1^	13 (12–17)	13 (12–17)	14 (12–17)	13 (12–17)	0.95
Sex, *n* (%)^2^					
Male	251 (80.19%)	155 (83.33%)	35 (74.47%)	61 (76.25%)	0.23
Female	62 (19.81%)	31 (16.67%)	12 (25.53%)	19 (23.75%)	
Injury etiology, *n* (%)^2^					
Traumatic	207 (65.71%)	126 (67.74%)	34 (70.83%)	47 (58.02%)	0.22
Non-traumatic	108 (34.29%)	60 (32.26%)	14 (29.17%)	34 (41.98%)	
Injury level, *n* (%)^2^					0.07
Tetraplegia	112 (36.13%)	61 (33.70%)	27 (56.25%)	24 (29.63%)	
Paraplegia	198 (63.87%)	120 (66.30%)	21 (43.75%)	57 (70.37%)	
Injury completeness, *n* (%)^2^					
Motor complete	63 (20.26%)	48 (26.37%)	8(16.4%)	7 (8.64%)	<**0.01**
Other	248 (79.74%)	134 (73.63%)	40 (83.33%)	74 (91.36%)	
SCIM score, median (IQR)^1^	31 (19–58)	27 (15–46.5)	30 (21–59)	43 (30–74)	**<0.01**

We tested differences between three participating centers using either ^1^Krushkal-Wallis or ^2^Chi^2^ test.

Statistically significant *p*-values are in bold.

## Discussion

The SwiSCI biobank and other SCI biobank endeavors are crucial if we are to take advantage of the multi-omics progress in biomedical research and big data use in epidemiology and to develop better understanding of health and functioning post-injury. Herewith, we provide an overview of the SwiSCI biobank potential and how it could be used in basic research and clinical practice and discuss challenges in maintaining the Biobank.

The SwiSCI biobank allows needle-to-freezer standardization in biospecimen processing, quality control of data, maximum efficiency of sample use and immediate return of data (via direct link with the SwiSCI database). Cryopreservation of multiple aliquots per same individual (e.g., on average nine serum aliquots per person) allow us to reanalyze the samples if necessary, which is a key for reproducibility of research; whereas longitudinal sampling (beginning and end of rehabilitation) provide us with a unique opportunity to explore temporality of associations and explore causal inference. Finally, the biobank may help fighting the major challenge in SCI research: limited availability of study participants and possibility to provide confirmatory data sets, by providing a basis to build collaborative national and international projects. This may not only increase the quality of research but may also lower the burden on individuals with SCI. For instance, repetitive inclusion of SCI participants in multiple observational studies may be avoided if biosamples may be re-analyzed. Furthermore, to facilitate digitalization of the biobanking effort, multi-omics data, a fast-growing area also in SCI research, can be layered on the top of clinical and other data routinely collected within SwiSCI and other cohorts. To promote and facilitate the FAIR data principles (data has to be Findable, Accessible, Interoperable, and Reusable) it is essential to improve standardization, and harmonize meta data and sample quality management among SCI biobanks internationally.

Other SCI biobanks may find it useful and learn from certain limitations and challenges which the SwiSCI biobank was faced when we operationalized the concept. The first challenge is the presence of participation bias. As of 19th October 2023, participation rate was around 60%. Based on previous SwiSCI study report, older persons, women, persons with lower functional independence and those with NTSCI were less likely to participate in the SwiSCI inception cohort, while females and older persons were less likely to consent to the SwiSCI biobank [9]. This may be important in regard of generalizability of findings based on SwiSCI biobank samples.

Second, we have noted variations in samples collection/processing among the participating rehabilitation centers which is most likely driven by the integrated in clinical routine sampling for the SwiSCI study within the SPZ but not of the other rehabilitation centers as well as slight variations in processing procedures and equipment and the involvement of multiple personnel. For example, the CRR, which is the furthers rehabilitation center performs the immediate biosample processing on site and storage at −80°C until shipment which occurs on regular basis whereas REHAB sends the fresh biospecimen with an express courier to be processed in the SwiSCI biobank which leads to a delayed processing.

Third, the SwiSCI is prospective study integrating extensive data collection at five time points, recruitment and sampling may be challenging, thus, careful planning of resources is needed to provide continued support to SwiSCI study center.

## Conclusion

The SwiSCI biobank is a unique research platform serving as a basis for collaborative SCI research. As part of the SwiSCI study it has established governance and approval regulations and structures. It has a dedicated team linking the participating rehabilitation centers, collecting and managing of data and biosamples. Repeated biosample collection allows for longitudinal research and causal inference analysis. Cryopreservation of multiple aliquots per same study participant facilitates reproducibility of results and provides practical appoach in decreasing burden to study participants.

The SwiSCI biobank and its standard operating procedures has been awarded quality labels by the Swiss Biobanking Platform thus opening the avenue for levels of harmoinsation and standartisation with other existing and future SCI biobanks from all over the world.

## Data Availability

Current article provides a descriptive overview of biosamples cryopreserved within the SwiSCI biobank, and therefore, no data were generated/analyzed.
